# High CRP/PNI levels predict an unfavorable prognosis in severe fever with thrombocytopenia syndrome: A propensity score matching study

**DOI:** 10.1002/iid3.1184

**Published:** 2024-02-20

**Authors:** Chunxia Guo, Huan Wang, Xiaorong Wang, Shan Tian

**Affiliations:** ^1^ Department of Infectious Diseases, Union Hospital, Tongji Medical College Huazhong University of Science and Technology Wuhan People's Republic of China; ^2^ Department of Infectious Diseases, Jinyinhu Hospital, Tongji Medical College Huazhong University of Science and Technology Wuhan People's Republic of China; ^3^ Department of Respiratory and Critical Care Medicine, Union Hospital, Tongji Medical College Huazhong University of Science and Technology Wuhan People's Republic of China

**Keywords:** C‐reactive protein, mortality, prognostic nutritional index, propensity score matching analysis, severe fever with thrombocytopenia syndrome

## Abstract

**Background:**

This study aimed to identify a novel inflammatory index and construct a nomogram for predicting in‐hospital mortality due to severe fever with thrombocytopenia syndrome (SFTS).

**Methods:**

This cohort included 610 patients with SFTS hospitalized in Wuhan Union Hospital between March 2017 and November 2022. The ratio of C‐reactive protein (CRP) to the prognostic nutritional index (PNI) was calculated and used to reflect patients' inflammatory status. Propensity score matching (PSM) was utilized to balance confounding factors between the low‐ and high‐CRP/PNI groups. SFTS individuals from Jinyinhu Hospital were used as the validation cohort.

**Results:**

Patients with SFTS and high CRP/PNI were significantly correlated with a higher percentage of severe and critical SFTS types and higher in‐hospital mortality rates than those with low CRP/PNI. CRP/PNI was the potent risk indicator for in‐hospital mortality in individuals with SFTS. The CRP/PNI nomogram showed a good predictive value for in‐hospital mortality in patients with SFTS. After PSM, the predictive performance of CRP/PNI for 28‐day mortality was excellent. Finally, the CRP/PNI could still assess patients with SFTS at different risks based on SFTS data from another medical center.

**Conclusion:**

The CPR/PNI ratio exhibited a strong positive correlation with the SFTS disease type and could predict in‐hospital mortality in the early stages of SFTS. The CPR/PNI ratio could substantially help clinicians facilitate the early identification of patients with high‐risk SFTS and the timely initiation of intensive therapy.

## Graphical abstract

Our study highlights the usefulness of the C‐reactive protein (CRP) to prognostic nutritional index (PNI) ratio in predicting the in‐hospital mortality of severe fever with thrombocytopenia syndrome (SFTS) patients based on a cohort of 610 SFTS cases from Wuhan Union Hospital. Subsequent survival analysis demonstrates that CRP/PNI could well differentiate SFTS individuals with high risk from those with low risk. receiver operating characteristic (ROC) analysis unveils that CRP/PNI possesses nice predictive power for predicting the in‐hospital mortality of SFTS. Briefly, CRP/PNI is a reliable biomarker that can serve as a promising predictor for subjects with SFTS.

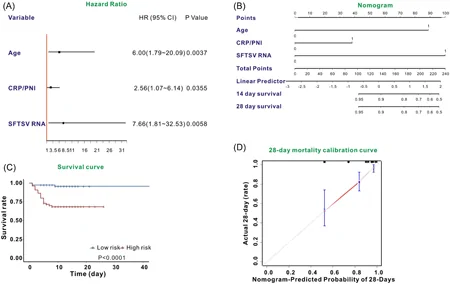

## 1 INTRODUCTION

Severe fever with thrombocytopenia syndrome (SFTS) is an emerging infectious disease caused by a novel bunyavirus.^1^ SFTS was first reported in China in 2009,^2^ and its mortality rate of up to 30% causes a heavy social burden. In 2018, the World Health Organization identified SFTS as a priority disease for medical research in emergency conditions. The incidence of SFTS is increasing worldwide and has become a global health concern because of the lack of effective vaccines.^3^ The typical manifestations of SFTS include fever, myocardial injury, nervous system symptoms, and thrombocytopenia. Owing to the lack of specific treatment agents,^4^ some cases of SFTS rapidly progress to multiple organ failure or even hemophagocytic syndrome, making SFTS a highly fatal infectious disease.^5^ Hence, identifying a reliable biomarker for the timely prediction of short‐term outcomes in patients with SFTS is vital for its clinical management.

The exact pathogenesis of SFTS is not completely understood but is reported to be correlated with excessive immune responses triggered by SFTS viral.^6^ Notably, the upregulation of natural killer (NK) cells leads to the excessive release of inflammatory cytokines, which may trigger a cytokine storm. Levels of interleukin (IL)‐10, IL‐6, monocyte chemoattractant protein‐1, tumor necrosis factor‐α, and granulocyte colony‐stimulating factor are upregulated in patients with SFTS, and the level of serum IL‐10 increases sharply in the early stage of SFTS.^7^ Moreover, the levels of the macrophage inflammatory proteins IL‐1 and IL‐8 are also increased in patients with severe SFTS.^7^ Changes in serum cytokine levels occupy a significant role in the SFTS progression and are correlated with worse clinical manifestations, such as renal failure^8^ and liver fibrosis.^9^ A recent study demonstrated that BCL2 antagonist/killer overexpression is associated with disease progression and in‐hospital mortality in patients with SFTS.^10^ Disturbance of the immune system triggered by SFTSV leads to excessive secretion of inflammatory factors, causing damage to multiple systems. Therefore, an accurate estimation of the inflammatory status of patients with SFTS is vital for risk assessment and survival prediction.

C‐reactive protein (CRP) is an acute‐phase inflammatory protein secreted by hepatocytes and is regulated by some cytokines. A recent clinical trial^11^ revealed that the serum CRP concentration in patients with SFTS was elevated compared to that in the controls. Further, extensive tissue damage caused by a high load of severe fever with thrombocytopenia syndrome virus (SFTSV) resulted in a significant upregulation of serum CRP concentration. Serum CRP levels can be used to differentiate SFTS from Japanese spotted fever with 95% sensitivity and 97% specificity.^12^ Chen et al.^13^ analyzed the clinical data of 256 patients with SFTS and concluded that serum CRP could moderately predict the in‐hospital mortality of patients with SFTS, with an area under the curve (AUC) > 0.7.

The prognostic nutritional index (PNI), initially designed to assess the surgical risk for patients with gastrointestinal tumors, consists of serum albumin and absolute lymphocyte count.^14^ Additionally, PNI could be used to assess the survival outcomes of patients with infectious diseases from the perspective of immune status.^15^ Generally speaking, the predictive performance of the joint index is higher than any single index in clinical practice. Hence, the CRP‐to‐PNI ratio (CRP/PNI) represents the inflammatory and immune statuses of the body. However, the combined use of inflammatory biomarkers in patients with SFTS has not been reported. Therefore, this study investigated whether CRP/PNI can be utilized as a reliable biomarker to assess the survival outcomes of patients with early‐stage SFTS. We also attempted to develop an easy‐to‐use SFTS nomogram for prognostic prediction to provide a reference for optimizing the allocation of healthcare resources for patients with SFTS.

## 2 MATERIALS AND METHODS

### 2.1 Study design

This clinical observational study was conducted at an accredited medical center (Wuhan Union Hospital) in Wuhan, China. This retrospective analysis included 610 patients with confirmed SFTS diagnoses from the Union Hospital of Tongji Medical College from March 2017 to December 2022. The inclusion criteria were as follows: (1) patients with fever and thrombocytopenia, (2) epidemiological history, (3) age >18 years, and (4) confirmed SFTSV using polymerase chain reaction. Patients with (1) active autoimmune diseases, (2) acute infection with other viruses, such as SARS‐CoV‐2 and epidemic hemorrhagic fever virus, and (3) missing critical information were excluded from the study. The clinical research plan was reviewed and approved by the Institutional Review Board of the Tongji Medical College (No. 2023‐S093). The clinical analysis was performed in accordance with the Declaration of Helsinki. The requirement for written informed consent was waived due to retrospective anonymized data collection. To verify the prognostic significance of CRP/PNI, clinical data of patients with SFTS during July 2021 and October 2023 with the same inclusion criteria from Jinyinhu Hospital, an affiliated hospital to the Tongji Medical College of Huazhong University of Science and Technology, were collected and analyzed.

### 2.2 Collection of clinical data

Generally, when the SFTS patients were admitted to our hospital, they would be prepared for drawing blood for examination at the time of admission if they were in bad condition. Otherwise, they would be prepared for drawing blood for examination on the next morning if they were in stable condition. The following clinical information was collected from the electronic medical records of the patients with SFTS: demographic features, vital signs, epidemiological history, clinical symptoms, length of hospital stay, and laboratory results. We collected the results of the first laboratory test within 24 h, including inflammatory indices (CRP and ferritin), blood routine (white blood cells [WBCs], monocytes, platelets, red blood cells, hemoglobin, eosinophils, lymphocytes, and basophils), liver function (alanine aminotransferase, aspartate aminotransferase, total bilirubin, and direct bilirubin), blood coagulation factors (activated partial thromboplastin time, d‐dimer, prothrombin time, and international normalized ratio), electrolytes (sodium, magnesium [Mg], calcium [Ca], and phosphorus), renal function (creatinine and blood urea nitrogen), and heart function (high‐sensitivity [hs] troponin and B‐type natriuretic peptide). All serum indices were converted into categorical data based on the normal ranges from our hospital. PNI was calculated as albumin (g/L) + 5 × lymphocyte count (10^9^/L). CRP/PNI was calculated as CRP/(albumin [g/L] + 5 × lymphocyte count [10^9^/L]). The 610 patients were divided into low and high CRP/PNI groups according to the median value of CRP/PNI (0.089) in the Union cohort. The SFTS disease types were divided into four groups (mild, common, severe, and critical) according to a recent consensus issued by the Chinese Society of Infectious Diseases.^16^ The endpoint of this clinical study was the in‐hospital 28‐day mortality rate.

### 2.3 Propensity score matching (PSM) analysis

As the division of the two groups (low vs. high CRP/PNI group) was not randomized in the primary SFTS cohort, an imbalance in some clinical features between the low and high CRP/PNI groups was observed. Therefore, PSM analysis was performed to balance these clinical variables between the low and high CRP/PNI groups, which could reduce the potential bias to some extent. A 1:1 (high vs. low CRP/PNI group) matched analysis with a nearest‐neighbor matching algorithm was performed to balance the confounding variables between the low and high CRP/PNI groups. The propensity score is defined to be the probability of exposure to the treatment conditional on a subject's observed baseline characteristics. The propensity score in our analysis was measured involving 19 clinical variables, including neutrophils, alanine aminotransferase, sodium, fibrinogen, procalcitonin, activated partial thromboplastin time, total bilirubin, blood urea nitrogen, red blood cell, total protein, monocyte, international normalized ratio, creatinine, platelet, white blood cell, lymphocyte, gamma‐glutamyltransferase, albumin, and phosphorus. The 1:1 PSM method yielded matched pairs of 77 SFTS subjects with low CRP/PNI and 77 patients with high CRP/PNI, with no differences between the two groups in the 19 covariates. PSM analysis using the matching package in the R software (version 3.4) generated a balanced SFTS cohort, including low (*N* = 77) and high (*N* = 77) CRP/PNI groups.

### 2.4 Statistical analysis

All statistical analyses were performed using SPSS (version 18.0) and R software (version 3.4). Enumeration indices, such as sex, are shown as the number of cases with percentages, and comparisons between the low and high CRP/PNI groups were performed using the χ^2^ or Fisher's exact test. Continuous data were expressed as mean ± standard deviation for normally distributed data, and the difference between the low and high CRP/PNI groups was detected using the Student's *t* test. Continuous data that did not conform to a normal distribution were presented as medians with corresponding interquartile ranges and were analyzed using nonparametric tests. Univariate and multivariate Cox regression analyses were performed to determine whether CRP/PNI was an independent risk factor for unfavorable survival outcomes in patients with SFTS. The independent risk variables identified using multivariate Cox regression were selected to construct the SFTS nomogram. Kaplan–Meier curves were generated to compare the in‐hospital mortality between the low and high CRP/PNI groups, and the difference in in‐hospital mortality between the low and high CRP/PNI groups was statistically measured using the log‐rank test. We also used receiver operating characteristic (ROC) curve analyses to quantify the predictive power of CRP/PNI and the prognostic nomogram for predicting the death rate of patients with SFTS. *p* < .05 at both sides was considered statistically significant.

## 3 RESULTS

### 3.1 Basic information of patients with SFTS

**Figure 1:**
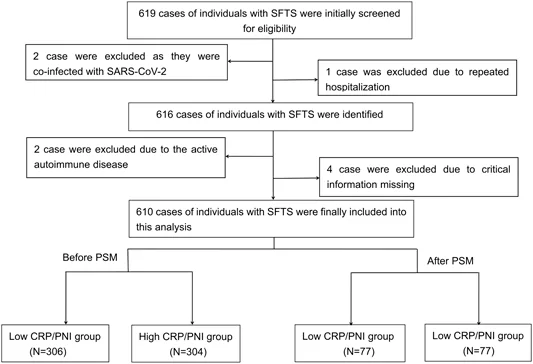
The detailed selection flow of SFTS individuals from the Wuhan Union cohort.

A total of 610 patients with SFTS from the Union cohort were enrolled in our clinical analysis. The detailed integration process is shown in Figure 1. The median age of the 610 patients with SFTS was 61.47 years. Among them, approximately 42.30% (*N* = 258) and 57.70% (*N* = 352) were female and male, respectively. A total of 19.02% (*N* = 116) and 12.30% (*N* = 75) patients with SFTS (19.02%) had a history of smoking and alcohol consumption, respectively. Among the 610 SFTS patients, 114 cases were diagnosed with hypertension, 56 cases were diagnosed with type 2 diabetes (T2DM), 33 cases were diagnosed with chronic obstructive pulmonary disease (COPD) and 68 cases were diagnosed with other diseases, such as chronic kidney disease, chronic liver disease, and coronary heart disease. The median time from illness onset to hospitalization is 5 (days) with the range of 1–7 (days). According to the classification criteria for SFTS, 267 patients were diagnosed with mild and moderate types of SFTS, and 343 patients were diagnosed with severe and critical types of SFTS. Our cohort included 81 deceased patients, with a mortality rate of 13.28%. Other clinical features of 610 patients with SFTS are listed in Table 1. Notably, the proportion of patients with SFTS who had high CRP/PNI was significantly higher among those who decreased from SFTS than those who had low CRP/PNI.

**Table 1:** Baseline features of SFTS patients in the primary cohort.

Index	Groups	Recovered (*n*%)	Decreased (*n*%)	*χ* ^2^ value	P value
Sex	Female	224 (42.34)	34 (41.98)		
	Male	305 (57.66)	47 (58.02)	0.0039	0.9501
Age	≤60 years	245 (46.31)	10 (12.35)		
	>60 years	284 (53.69)	71 (87.65)	33.3154	<0.0001
Smoking	No	248 (70.66)	26 (66.67)		
	Yes	103 (29.34)	13 (33.33)	0.2672	0.6052
Alcohol	No	270 (80.60)	28 (73.68)		
	Yes	65 (19.40)	10 (26.32)	1.0152	0.3137
CRP/PNI	Low	282 (53.31)	24 (29.63)		
	High	247 (46.69)	57 (70.37)	15.7537	<0.0001
SFTSV RNA	Low	296 (56.49)	9 (11.25)		
	High	228 (43.51)	71 (88.75)	56.8204	<0.0001
CRP	Normal	327 (61.81)	17 (20.99)		
	High	202 (38.19)	64 (79.01)	47.6132	<0.0001
PCT	Normal	220 (43.31)	10 (13.16)		
	High	288 (56.69)	66 (86.84)	25.1716	<0.0001
WBC	Normal	178 (33.65)	18 (22.22)		
	Abnormal	351 (66.35)	63 (77.78)	4.2055	0.0403
RBC	Normal	437 (83.40)	65 (80.25)		
	Low	87 (16.60)	16 (19.75)	0.4928	0.4827
HGB	Normal	393 (75.29)	61 (75.31)		
	Low	129 (24.71)	20 (24.69)	0	0.9967
PLT	>50 G/L	94 (17.77)	33 (40.74)		
	30–50 G/L	172 (32.51)	30 (37.04)		
	<30 G/L	263 (49.72)	18 (22.22)	29.5155	<0.0001
Lym	Normal	141 (26.91)	14 (17.28)		
	Abnormal	383 (73.09)	67 (82.72)	3.4102	0.0648
Mon	Normal	293 (57.00)	29 (37.18)		
	Abnormal	221 (43.00)	49 (62.82)	10.729	0.0011
Neu	Normal	198 (37.50)	30 (37.04)		
	Abnormal	330 (62.50)	51 (62.96)	0.0064	0.9361
ALT	Normal	67 (12.67)	8 (9.88)		
	High	462 (87.33)	73 (90.12)	0.5067	0.4766
AST	Normal	17 (3.21)	1 (1.23)		
	High	512 (96.79)	80 (98.77)	0.9607	0.327
GGT	Normal	247 (46.69)	31 (38.27)		
	High	282 (53.31)	50 (61.73)	2.0079	0.1565
ALP	Normal	455 (87.00)	67 (83.75)		
	High	68 (13.00)	13 (16.25)	0.6295	0.4275
TBIL	Normal	475 (89.96)	68 (83.95)		
	High	53 (10.04)	13 (16.05)	2.6264	0.1051
TP	Normal	174 (33.14)	26 (32.50)		
	Low	351 (66.86)	54 (67.50)	0.013	0.9093
ALB	Normal	117 (22.12)	14 (17.28)		
	Low	412 (77.88)	67 (82.72)	0.9731	0.3239
GLB	Normal	414 (79.01)	58 (72.50)		
	High	110 (20.99)	22 (27.50)	1.721	0.1896
CREA	Normal	419 (86.39)	48 (62.34)		
	High	66 (13.61)	29 (37.66)	27.372	<0.0001
BUN	Normal	373 (71.32)	47 (58.02)		
	High	150 (28.68)	34 (41.98)	5.8521	0.0156
LDH	Normal	5 (0.96)	0 (0.00)		
	High	516 (99.04)	81 (100.00)	0.7839	0.376
CK	Normal	71 (13.63)	2 (2.47)		
	High	450 (86.37)	79 (97.53)	8.1913	0.0042
Hs_trop	Normal	78 (18.35)	2 (2.70)		
	High	347 (81.65)	72 (97.30)	11.4672	0.0007
CK‐MB	Normal	307 (78.72)	35 (53.03)		
	High	83 (21.28)	31 (46.97)	19.8651	<0.0001
Ferrin	Normal	2 (0.60)	0 (0.00)		
	High	332 (99.40)	43 (100.00)	0.2589	0.6109
Na	Normal	282 (54.23)	36 (45.57)		
	Low	238 (45.77)	43 (54.43)	2.0657	0.1506
Ka	Normal	523 (6)	79 (2)	8.9138	0.0028
	Low	298 (56.98)	59 (74.68)		
Ca	Normal	144 (27.75)	9 (11.54)		
	Low	375 (72.25)	69 (88.46)	9.345	0.0022
Mg	Normal	348 (68.37)	41 (53.95)		
	Low	161 (31.63)	35 (46.05)	6.1735	0.013
Phosphorus	Normal	183 (35.60)	31 (39.74)		
	Low	331 (64.40)	47 (60.26)	0.503	0.4782
APTT	Normal	140 (26.87)	3 (3.75)		
	High	381 (73.13)	77 (96.25)	20.447	<0.0001
D_dimer	Normal	10 (2.08)	0 (0.00)		
	High	470 (97.92)	78 (100.00)	1.6547	0.1983
FIB	Normal	441 (84.81)	60 (75.00)		
	High	79 (15.19)	20 (25.00)	4.8407	0.0278
INR	Normal	489 (94.22)	62 (78.48)		
	High	30 (5.78)	17 (21.52)	23.452	<0.0001
PT	Normal	461 (89.00)	56 (70.00)		
	High	57 (11.00)	24 (30.00)	21.3538	<0.0001
TT	Normal	108 (20.77)	6 (7.50)		
	High	412 (79.23)	74 (92.50)	7.9322	0.0049
Disease type	Mild	61 (11.53)	0 (0.00)		
	Moderate	202 (38.19)	4 (4.94)		
	Severe	172 (32.51)	38 (46.91)		
	Critical	94 (17.77)	39 (48.15)	61.7433	<0.0001

Abbreviations: ALB, albumin; ALP, alkaline phosphatase; ALT, alanine aminotransferase; APTT, activated partial thromboplastin time; AST, aspartate aminotransferase; BUN, blood urea nitrogen; CK, creatine kinase; CK‐MB, creatine kinase‐myoglobin binding; CREA, creatinine; CRP, C‐reactive protein; FIB, fibrinogen; GGT, gamma glutamyl transferase; GLB, globulin; HGB, hemoglobin; INR, international normalized ratio; K, potassium; LDH, lactate dehydrogenase; Mg, magnesium; Mon, monocyte; Na, sodium; Neu, neutrophil count; PCT, procalcitonin; PLT, platelet count; PNI, prognostic nutritional index; PSM, propensity score matching; PT, prothrombin time; RBC, red blood cells; SFTS, severe fever with thrombocytopenia syndrome; SFTSV, severe fever with thrombocytopenia syndrome virus; TBIL, total bilirubin; TP, total protein; TT, thrombin time; WBC, white blood cells.

### 3.2 Correlation between CRP/PNI and clinical features

**Figure 2:**
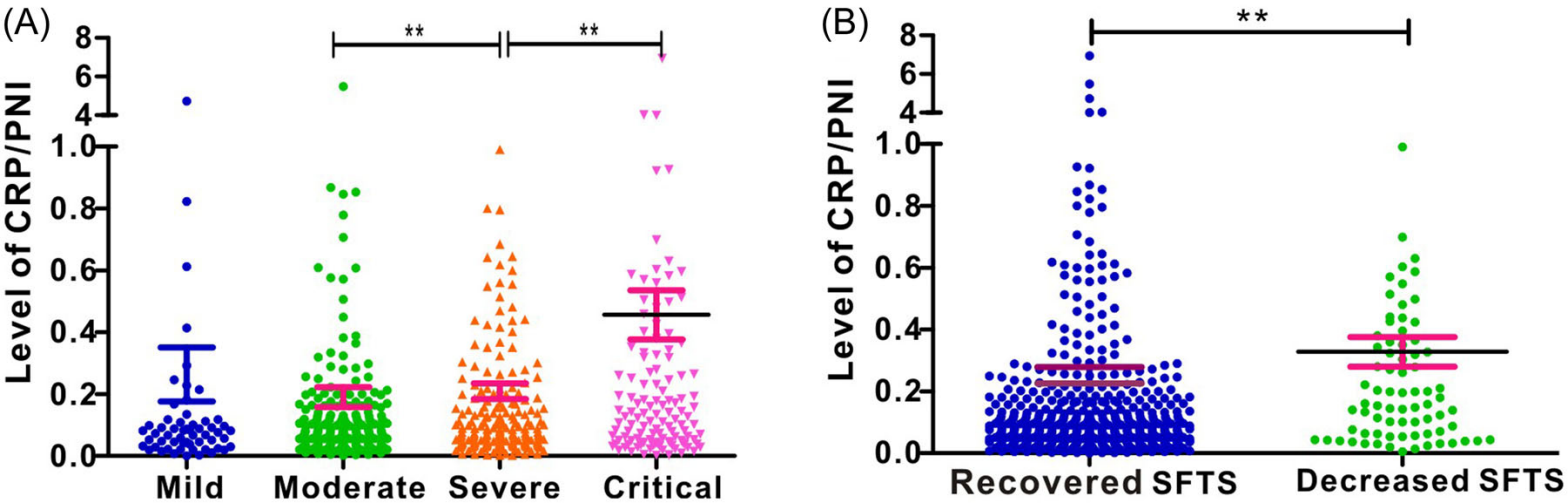
The levels of CRP/PNI in SFTS patients with different disease types and living status. (A) The levels of CRP/PNI in four disease types of SFTS. (B) The levels of CRP/PNI were also higher in deceased SFTS individuals compared with those recovered. CRP, C‐reactive protein; PNI, prognostic nutritional index; SFTS, severe fever with thrombocytopenia syndrome.

A total of 610 patients were divided into low (*N* = 306) and high (*N* = 304) CRP/PNI groups according to the median value of CRP/PNI (0.089) in the entire SFTS cohort. We then used the χ^2^ test or Fisher's exact analysis to measure the differences in clinical metrics between the low and high CRP/PNI groups. As listed in Supporting Information S1: Table S1, CRP/PNI levels significantly correlated with most clinical features of SFTS, including WBCs (*p* < .001), procalcitonin (*p* = .0008), platelet (*p* = .027), neutrophils (*p* < .0001), and activated partial thromboplastin time (*p* = .0011). The levels of CRP/PNI were significantly different via the one‐way analysis of variance among the four SFTS disease types (*p* = .0009), and the difference in CRP/PNI was significant between the moderate and severe types (*p* = .0006; Figure 2A) and between the severe and critical types (*p* = .0005; Figure 2A). Importantly, CRP/PNI levels were also higher in deceased patients compared with those who recovered from SFTS (*p* < .0001; Figure 2B).

### 3.3 Survival analysis and predictive significance of CRP/PNI

**Figure 3:**
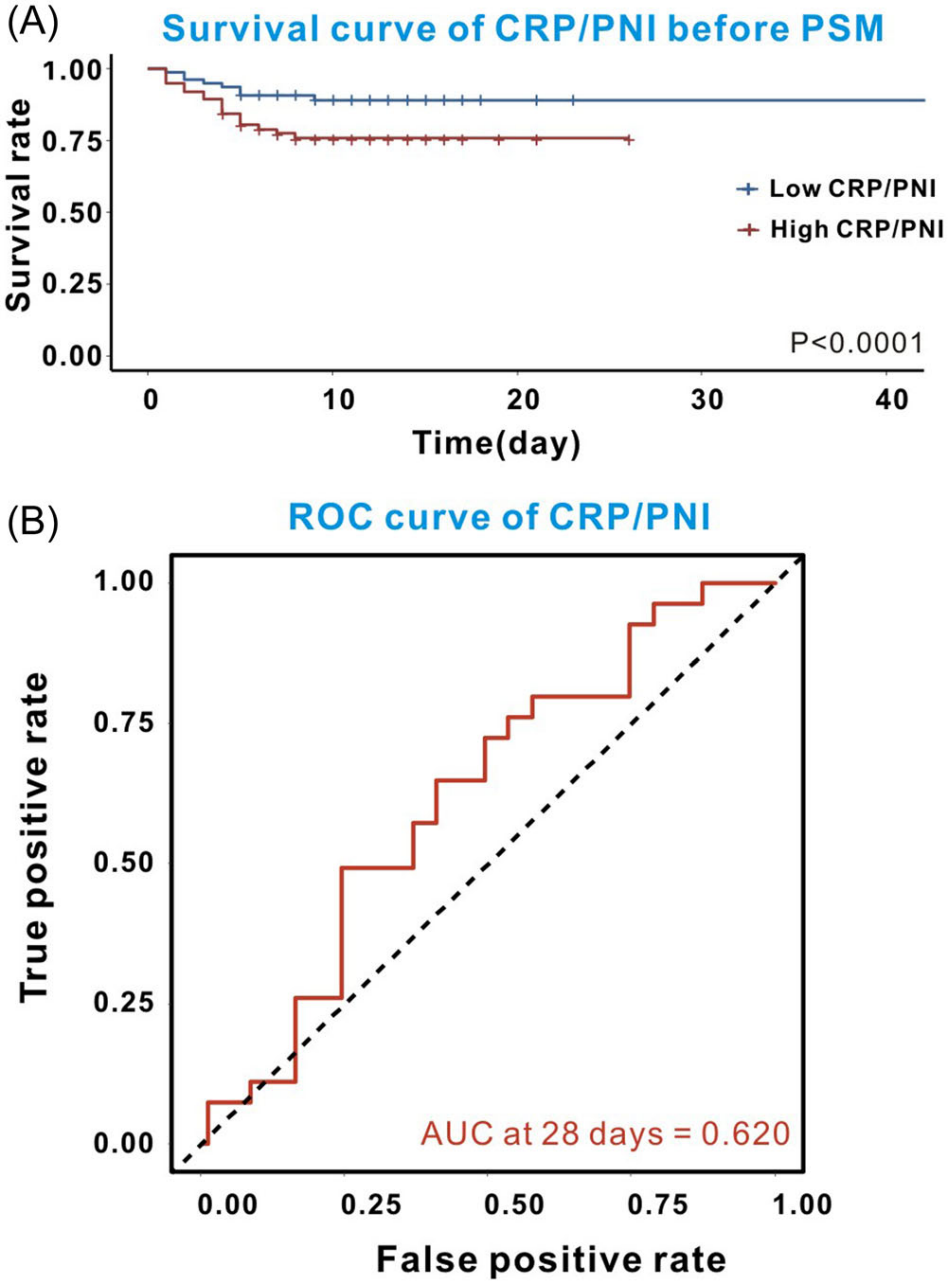
Prognostic significance and predictive ability of CRP/PNI in SFTS individuals before propensity matching. (A) SFTS individuals with high levels of CRP/PNI exhibited an increased risk of in‐hospital death compared to those with low levels of CRP/PNI before propensity matching. (B) ROC curve analysis demonstrated that the level of CRP/PNI could well predict the in‐hospital 28‐day mortality among SFTS individuals before propensity matching. AUC, area under the curve; CRP, C‐reactive protein; PNI, prognostic nutritional index; ROC, receiver operating characteristic; SFTS, severe fever with thrombocytopenia syndrome.

Kaplan–Meier curves were plotted to determine whether there was any difference in in‐hospital mortality between the low and high CRP/PNI groups. The log‐rank test revealed that patients with SFTS in the low CRP/PNI group exhibited a lower risk of in‐hospital death (hazard ratio [HR] = 2.550, 95% confidence interval [CI]: 1.583–4.109, *p* < .0001) compared to those in the high CRP/PNI group (Figure 3A). The ROC was used to assess the power of CRP/PNI in predicting the survival outcome of patients with SFTS. As shown in the ROC curve, CRP/PNI had a good ability to predict 28‐day mortality in patients with SFTS with an AUC of 0.62 (Figure 3B).

### 3.4 Survival nomogram incorporating CRP/PNI

**Figure 4:**
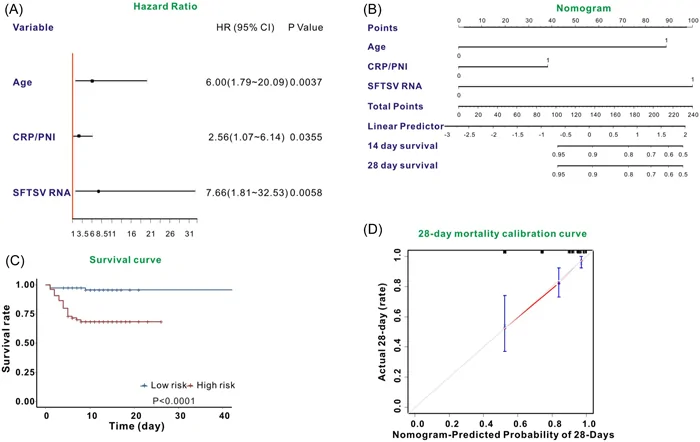
The prognostic value and calibration ability of prognostic nomogram incorporating CRP/PNI in SFTS individuals before propensity matching. (A) Forest plot of independent risk factors via multivariate Cox regression before propensity matching. (B) The prognostic nomogram incorporating CRP/PNI before propensity matching. (C) SFTS individuals with high risk exhibited an increased risk of in‐hospital mortality compared to those with low risk before propensity matching. (D) The calibration curve indicated that the 28‐day mortality predicted by the SFTS nomogram incorporating CRP/PNI was almost consistent with the actual 28‐day in‐hospital mortality before propensity matching. CRP, C‐reactive protein; PNI, prognostic nutritional index; SFTS, severe fever with thrombocytopenia syndrome.

Univariate Cox regression analysis demonstrated that patients with SFTS with high CRP/PNI, older age, high SFTSV RNA load, increased creatine kinase (CK), increased hs troponin, elevated CK‐MB, low Mg, low Ca, long PT, and long thrombin time were all risk factors for in‐hospital death of patients with SFTS. Subsequently, these significant variables derived using the univariate Cox regression analysis were selected for multivariate Cox regression (Supporting Information S1: Table S2). The result from multivariate Cox regression indicated that high CRP/PNI (HR = 2.248, 95% CI: 1.3–3.889, *p* = .0038), high SFTSV RNA load (HR = 5.905, 95% CI: 2.783–12.529, *p* < .0001), increased creatine kinase‐myoglobin binding (CK‐MB) (HR = 2.549, 95% CI: 1.516–4.285, *p* = .0004), older age (HR = 4.702, 95% CI: 2.223–9.945, *p* < .0001), and long prothrombin time (PT) (HR = 2.214, 95% CI: 1.273–3.851, *p* = .0049) could independently affect the in‐hospital death of patients with SFTS (Figure 4A). We then constructed a survival nomogram based on the five significant prognostic indicators (Figure 4B). The prognostic nomogram based on CRP/PNI could stratify patients with SFTS into low‐ and high‐risk groups (Figure 4C). Moreover, calibration curves revealed that the predicted survival rates via the SFTS nomogram better reflected the actual 28‐day (Figure 4D) survival probabilities of patients with SFTS. Finally, the nomogram based on CRP/PNI could well predict the 28‐day mortality, with an AUC of 0.885 (Supporting Information S1: Figure S1A).

### 3.5 PSM analysis

We used a 1:1 PSM to reduce confounders between the low and high CRP/PNI groups. After PSM, 77 patients with SFTS were included in the low CRP/PNI group, and 77 patients with SFTS were included in the high CRP/PNI group in the PSM cohort. The baseline features of the patients with SFTS are listed in Supporting Information S1: Table S3. After PSM, the clinical features were more balanced between the low and high CRP/PNI groups (Supporting Information S1: Table S4). In the PSM cohort, patients with SFTS with low CRP/PNI had decreased 28‐day mortality compared to those with high CRP/PNI (Supporting Information S1: Figure S2A). As shown in the ROC curve, on admission, CRP/PNI had good ability, with an AUC of 0.85, to predict 28‐day in‐hospital mortality for patients with SFTS (Supporting Information S1: Figure S2B).

**Figure 5:**
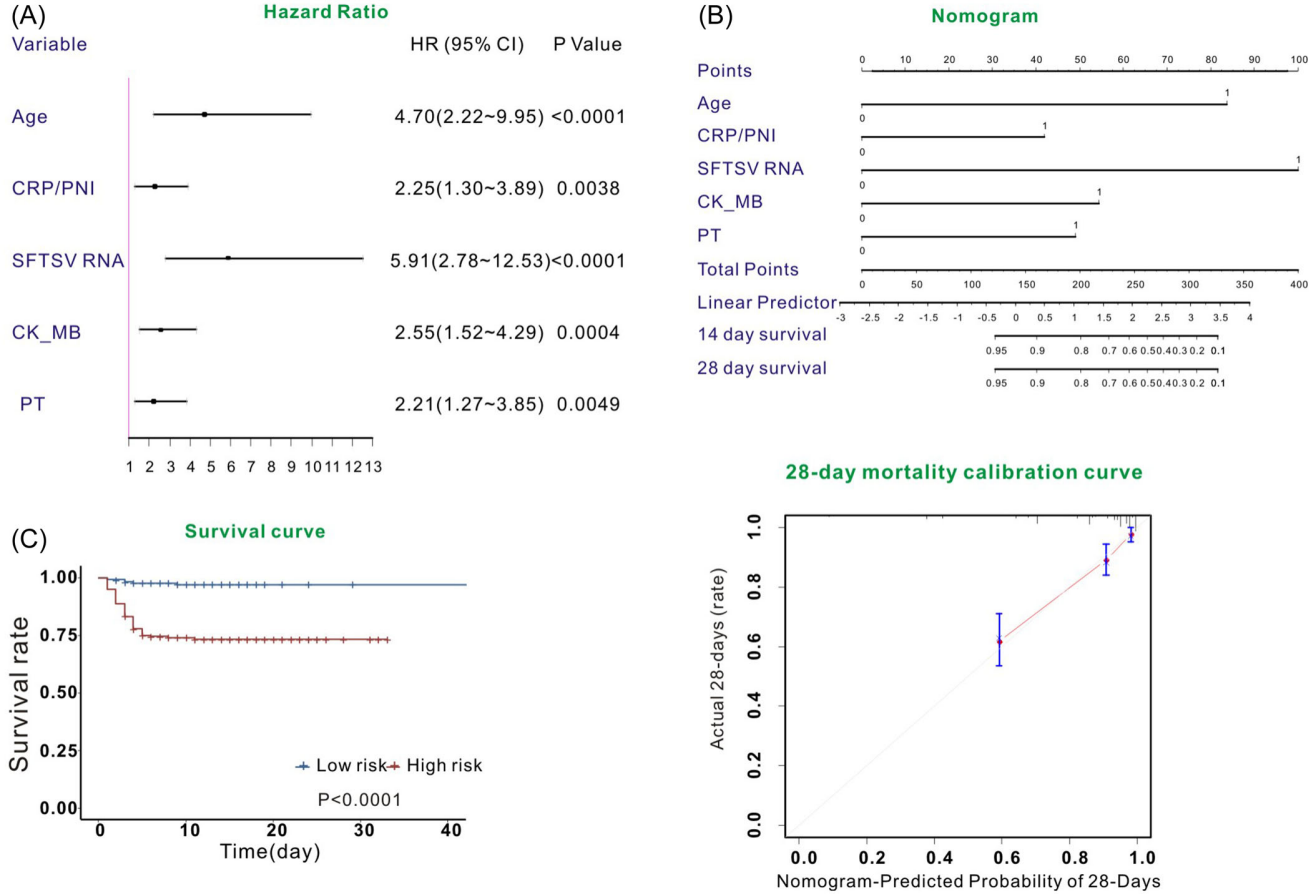
The prognostic value and calibration ability of prognostic nomogram incorporating CRP/PNI in SFTS individuals from the propensity matching cohort. (A) Forest plot of independent risk factors via multivariate Cox regression after propensity matching. (B) The prognostic nomogram incorporating CRP/PNI after propensity matching. (C) SFTS individuals with high risk exhibited an increased risk of in‐hospital death compared to those with low risk in the propensity matching cohort. (D) The calibration curve indicated that the 28‐day mortality predicted by the SFTS nomogram incorporating CRP/PNI was almost consistent with the actual 28‐day mortality in the propensity‐matching cohort. CRP, C‐reactive protein; PNI, prognostic nutritional index; SFTS, severe fever with thrombocytopenia syndrome.

Univariate Cox regression in the PSM cohort demonstrated that high CRP/PNI, older age, high SFTSV RNA load, decreased Mg, and long PT were all risk factors for in‐hospital death in patients with SFTS. Subsequently, the significant variables derived from the univariate Cox regression were used in the multivariate Cox regression analysis (Supporting Information S1: Table S5). The results from multivariate Cox regression indicated high levels of CRP/PNI (HR = 2.557, 95% CI: 1.066–6.137, *p* = .0355), high SFTSV RNA load (HR = 7.662, 95% CI: 1.805–32.532, *p* = .0058), and older age (HR = 5.96, 95% CI: 1.79–20.09, *p* = .0037) could independently affect the in‐hospital death of patients with SFTS (Figure 5A). We then constructed a survival nomogram based on three significant prognostic indicators (Figure 5B). The prognostic nomogram based on CRP/PNI after PSM could stratify patients with SFTS into low‐ and high‐risk groups (Figure 5C). Moreover, calibration curves revealed that the predicted survival rates via the SFTS nomogram better reflected the actual 28‐day (Figure 5D) survival probabilities of patients with SFTS. Finally, the nomogram based on CRP/PNI could well predict the 28‐day mortality, with an AUC of 0.936 (Supporting Information S1: Figure S1B).

### 3.6 External validation of the prognostic performance of CRP/PNI

**Figure 6:**
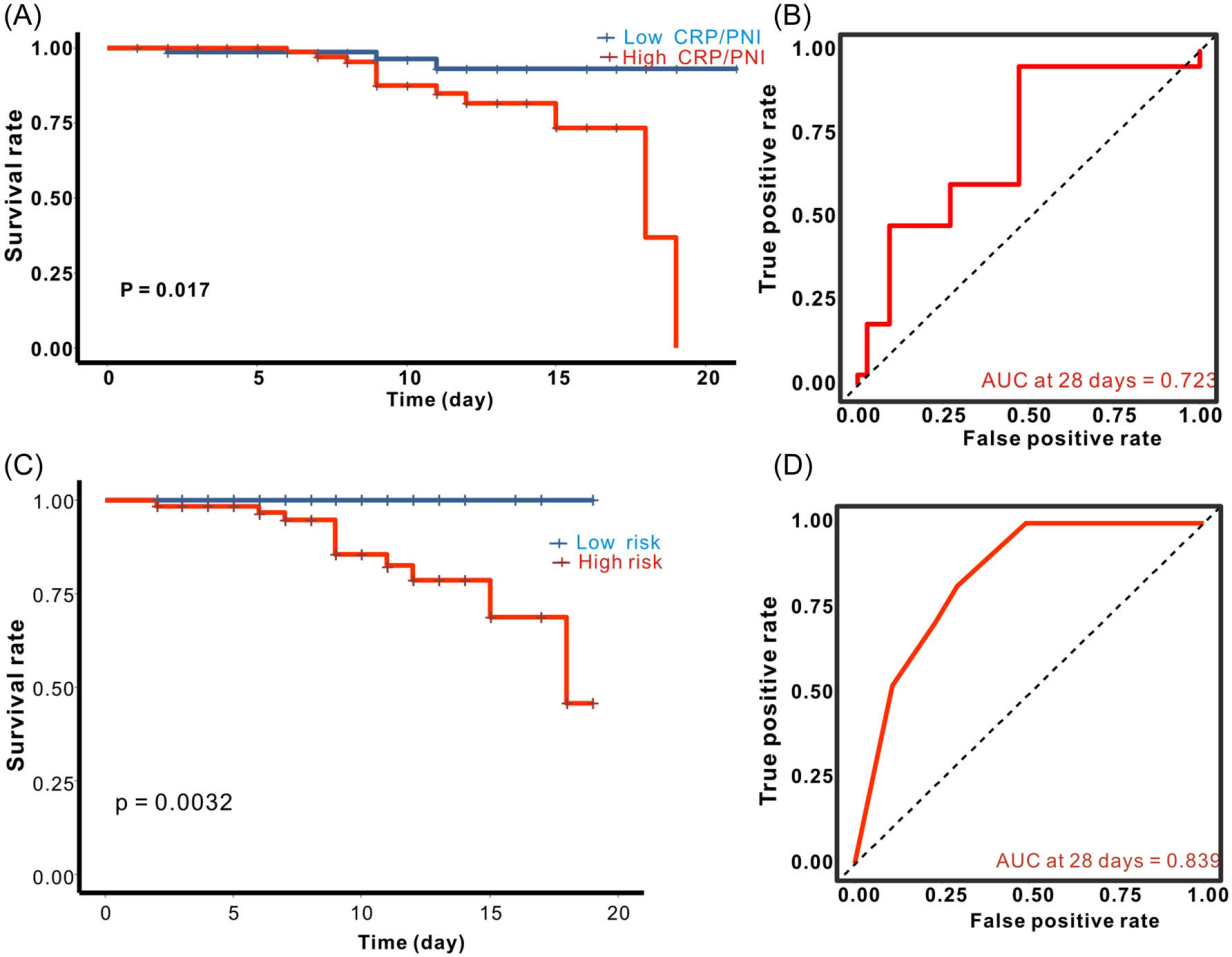
External validation of CRP/PNI in Jinyinhu cohort. (A) Survival analysis of CRP/PNI. (B) Receiver operating characteristic analysis of CRP/PNI. (C) Survival analysis of SFTS nomogram. (D) Receiver operating characteristic analysis of SFTS nomogram. CRP, C‐reactive protein; PNI, prognostic nutritional index; SFTS, severe fever with thrombocytopenia syndrome.

A total of 137 patients with SFTS who met the same inclusion criteria as the Jinyinhu cohort were included for external verification. Among them, 57 and 80 were male and female, respectively. Notably, 15 patients died of SFTS, and the approximate mortality rate of SFTS in the Jinyinhu cohort was 10.95%. Survival analysis using SFTS data from the Jinyinhu cohort showed that patients with SFTS and low CRP/PNI had shorter survival time than those with high CRP/PNI (*p* = .017, Figure 6A). ROC analysis revealed that the predictive performance of CRP/PNI for 28‐day mortality is 0.723 (Figure 6B). Moreover, the SFTS nomogram including CR/PNI also stratified SFTS from the Jinyinhu cohort into a high‐risk group and a low‐risk group, and survival analysis demonstrated that SFTS patients with low risk exhibited better survival outcome than those with high risk (*p* = .0032, Figure 6C). The SFTS nomogram including CRP/PNI obtained nice predictive accuracy (AUC: 0.835, Figure 6D) in the Jinyinhu cohort. Hence, we concluded that CRP/PNI is a reliable risk score for the prognostic prediction of patients with SFTS.

## 4 DISCUSSION

SFTS is a life‐threatening infectious disease with a high mortality rate. Early identification of patients with SFTS with a high mortality risk and intensive treatment, such as glucocorticoids and gamma globulin, may decrease the in‐hospital death rate. The CRP‐to‐PNI ratio, which is derived from CRP, serum albumin, and lymphocytes, is a combined biomarker for the assessment of systemic inflammation and immune status. For the first time, our study highlights the usefulness of the CRP‐to‐PNI ratio in predicting the in‐hospital mortality of patients with SFTS based on a cohort of 610 patients with SFTS from Wuhan Union Hospital. Survival analysis demonstrated that CRP/PNI could differentiate patients with high‐risk SFTS from those with low‐risk SFTS. ROC analysis revealed that CRP/PNI possesses good predictive power for in‐hospital mortality in patients with SFTS. Subsequent external validation of the Jinyinhu cohort also highlighted a close correlation between CRP/PNI levels and mortality among patients with SFTS. Briefly, CRP/PNI is a reliable biomarker that can serve as a promising predictor in patients with SFTS.

Albumin, secreted by the liver, is the main transport protein in blood and is a vital nutrient for the human body. Albumin is often used to measure nutritional status in clinical settings. Albumin is closely associated with the inflammatory response, and systemic inflammation accelerates the breakdown of serum albumin.^17^ Low serum albumin levels reflect impaired liver function during infectious diseases, as inflammatory factors can impede albumin synthesis.^18^ A previous report indicated that low levels of serum albumin are closely correlated with high in‐hospital mortality among patients with SFTS.^19^ The PNI, derived from serum albumin and lymphocyte count, is an objective reflection of the inflammatory status. The PNI has been widely investigated in COVID‐19 but has never been explored in SFTS. Our clinical analysis showed that the PNI was significantly different among patients with decreased SFTS and recovered SFTS.

SFTS is featured by thrombocytopenia syndrome and coagulopathy, and some patients are prone to spontaneous hemorrhage,^20^ which would cause hemoconcentration. Moreover, in some SFTS individuals with septic shock, excessive fluid replacement can cause blood dilution. The two conditions could directly infect the absolute value of CRP or serum albumin. The ratio of CRP to PNI is not influenced by hemoconcentration or blood dilution, which is more accurate than the absolute value of serum CRP or albumin. CRP/PNI, an objective inflammatory index, has been reported in cancer populations. Chen et al.^21^ identified CRP/PNI as a novel prognostic score for patients with esophageal squamous cell carcinoma. Fu and Yang^22^ demonstrated that the CRP/PNI is a prognostic indicator in patients with laryngeal cancer. Ren et al.^23^ found that a high CRP/PNI was closely associated with high in‐hospital mortality in older patients who underwent hip fracture surgery. In our analysis, the prognostic significance of the CRP/PNI among patients with SFTS was much better than that of the PNI or CRP alone. A high CRP/PNI signifies an excessive inflammatory status in patients with SFTS, which correlates with a higher risk of death. In addition, CRP/PNI with a cut‐off value of 0.089 could identify patients with high‐risk SFTS as early as at the time of admission within 24 h and aid clinicians in selecting timely and intensive treatment.

PSM analysis, a classical statistical method, is commonly used in clinical trials. PSM analysis is used to reduce the potential bias from observational cohorts rather than from randomized controlled cohorts. In addition, it removes the effects of confounding covariates by matching the case group with the control group, which exhibits a similar propensity.^24^ In our previous research,^25^ we used PSM analysis to balance the confounding bias between high and low Gustave Roussy Immune (GRIm)‐Score groups and concluded that the prognostic value of the GRIm‐Score among patients with colorectal cancer was more significant. Xiong et al.^26^ conducted a 1:1 PSM analysis to adjust the baseline differences between the corticosteroid‐ and noncorticosteroid‐treated groups and concluded that treatment with corticosteroids showed no impact on clinical outcome in patients with SFTS, and its use was associated with an increased risk of secondary infections. Jung et al.^27^ conducted a retrospective cohort study on SFTS and concluded that steroid therapy was associated with an increased risk of complications in patients with SFTS after adjusting for nine confounding variables. We found that the prognostic role of CRP/PNI in patients with SFTS is more robust after PSM analysis, and our conclusion based on the 1:1 PSM analysis was more reliable and rigorous. After PSM, the predictive ability of CRP/PNI for 28‐day mortality was stronger than before PSM.

To our knowledge, our sample size is the largest among previously published reports. A study on SFTS from the First Affiliated Hospital of Anhui Medical University included 160 patients with SFTS.^28^ Another multicenter cohort study from 13 university hospitals in Korea included 142 patients with SFTS.^27^ In addition, a study on SFTS conducted in Nanjing included 256 patients.^13^ Notably, our hospital was one of the earliest hospitals to discover this emerging infectious disease^29^ and has accumulated a large amount of experience in treating SFTS. Wuhan Union Hospital is the largest treatment center for SFTS in Central China. Hence, the sufficient sample size of patients with SFTS from our hospital lays a strong foundation for our reliable conclusions. Our study has two limitations. First, although we collected SFTS data from the Jinyinhu cohort for external validation, the sample size of patients with SFTS was relatively small (*N* = 138). Second, we only detected CRP/PNI on admission within 48 h but lacked dynamic monitoring for assessing this infectious disease. Hence, our future work will focus on the validation and refinement of the CRP/PNI in larger SFTS cohorts from different medical centers.

## 5 CONCLUSIONS

Our retrospective study indicates that the CPR/PNI ratio exhibits a strong positive correlation with the disease type of SFTS and could predict in‐hospital mortality in the early stage among patients with SFTS. The prognostic nomogram based on this novel biomarker could predict the 28‐day mortality among patients with SFTS. The CPR/PNI ratio can potentially help clinicians facilitate early identification of high‐risk SFTS and initiate intensive therapy, which might decrease the risk of death to some extent.

## AUTHOR CONTRIBUTIONS

Shan Tian and Xiaorong Wang designed the clinical study and took responsibility for the integrity and accuracy of the data. Chuanxia Guo contributed to the drafting of the manuscript and performed the data analysis. Huan Wang collected data and contributed to the data analysis. All authors read and approved the final version of this manuscript.

## CONFLICT OF INTEREST STATEMENT

The authors declare no conflict of interest.

## ETHICS STATEMENT

The clinical study was reviewed and successfully approved by the institutional review board of Tongji Medical College (No. 2023‐S093). The requirement for getting written informed consent was waived due to the retrospective anonymized data collection.

## Supporting information

Supporting information.

## Data Availability

The original data generated during this analysis are available from the corresponding author upon reasonable request.
