# Supplementary material for: High CRP/PNI levels predict an unfavorable prognosis in severe fever with thrombocytopenia syndrome: A propensity score matching study
Source: Immun Inflamm Dis. 2024 Feb 20;12(2):e1184. doi: 10.1002/iid3.1184 (PMC10877553; doi:10.1002/iid3.1184)
Supplement: Supplementary file 1 — Supporting information. [file IID3-12-e1184-s001.doc]

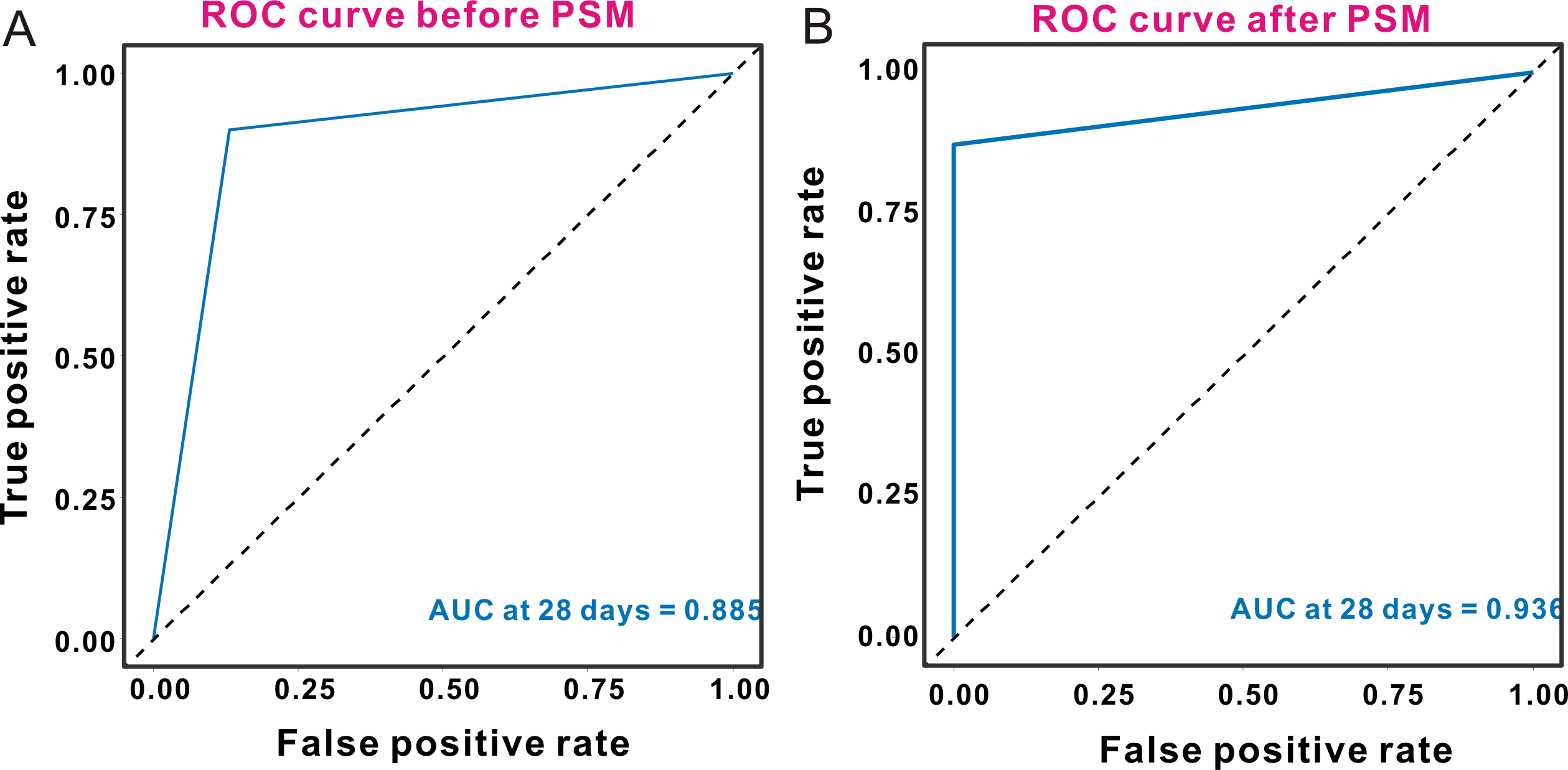


**Figure S1**. ROC curve analysis demonstrated that the SFTS nomogram incorporating CRP/PNI could well predict the in-hospital 28-day mortality both in the primary cohort (**A**) and PSM cohort( **B**).


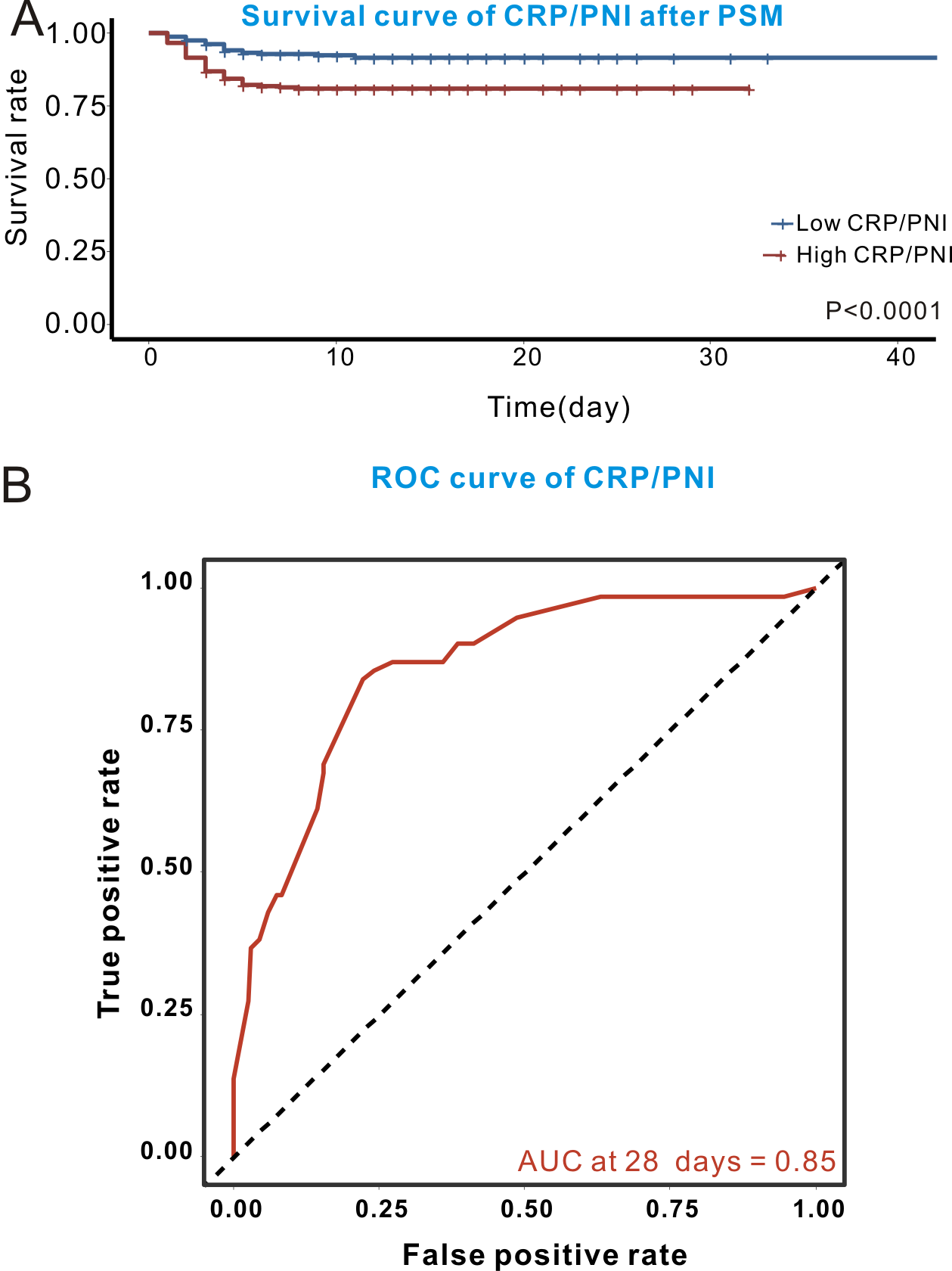


**Figure S2**. Prognostic significance and predictive ability of CRP/PNI in SFTS individuals after propensity matching. **A**. SFTS individuals with high level of CRP/PNI exhibited increased risk of mortality compared to those with low level of CRP/PNI after propensity matching. **B**. ROC curve analysis demonstrated that level of CRP/PNI could well predict the 28-day mortality among SFTS individuals after propensity matching.

**Table S1**. Clinical correlation of CRP/PNI in individuals with SFTS before PSM

| **Index** | **Groups** | **Low CRP/PNI** | **High CRP/PNI** | **X^2^ value** | **P value** |
| --- | --- | --- | --- | --- | --- |
| Sex | Female | 121(39.54) | 137(45.07) |  |  |
|  | Male | 185(60.46) | 167(54.93) | 1.9062 | 0.1674 |
| Age | ≤60 years | 139(45.42) | 116(38.16) |  |  |
|  | >60 years | 167(54.58) | 188(61.84) | 3.3102 | 0.0688 |
| smoking | No | 153(76.88) | 121(63.35) |  |  |
|  | Yes | 46(23.12) | 70(36.65) | 8.5422 | 0.0035 |
| alcohol | No | 165(86.39) | 133(73.08) |  |  |
|  | Yes | 26(13.61) | 49(26.92) | 10.2784 | 0.0013 |
| FSTSV RNA | Low | 255(83.33) | 89(29.28) |  |  |
|  | High | 51(16.67) | 215(70.72) | 181.2128 | <0.0001 |
| PCT | Normal | 60(19.61) | 136(44.74) |  |  |
|  | High | 246(80.39) | 168(55.26) | 44.159 | <0.0001 |
| WBC | Normal | 265(86.89) | 237(79.00) |  |  |
|  | Abnormal | 40(13.11) | 63(21.00) | 6.6568 | 0.0099 |
| RBC | Normal | 235(78.07) | 219(72.52) |  |  |
|  | Low | 66(21.93) | 83(27.48) | 2.5018 | 0.1137 |
| HGB | Normal | 2(0.65) | 0(0.00) |  |  |
|  | Low | 304(99.35) | 304(100.00) | 1.9935 | 0.158 |
| PLT | >50G/L | 149(48.69) | 132(43.42) | 5.1169 | 0.0237 |
|  | 30-50 | 17(5.59) | 138(45.85) |  |  |
|  | <30G/L | 287(94.41) | 163(54.15) | 128.6152 | <0.0001 |
| Mon | Normal | 92(30.07) | 136(44.88) |  |  |
|  | Abnormal | 214(69.93) | 167(55.12) | 14.2747 | 0.0002 |
| Neu | Normal | 154(50.33) | 137(45.07) | 1.6919 | 0.1933 |
|  | Abnormal | 53(17.32) | 22(7.24) |  |  |
| ALT | Normal | 253(82.68) | 282(92.76) | 14.3789 | 0.0001 |
|  | High | 11(3.59) | 7(2.30) |  |  |
| AST | Normal | 295(96.41) | 297(97.70) | 0.8891 | 0.3457 |
|  | High | 177(57.84) | 101(33.22) |  |  |
| GGT | Normal | 129(42.16) | 203(66.78) | 37.2648 | <0.0001 |
|  | High | 267(87.83) | 255(85.28) |  |  |
| ALP | Normal | 37(12.17) | 44(14.72) | 0.8394 | 0.3596 |
|  | High | 285(93.14) | 258(85.15) |  |  |
| TBIL | Normal | 21(6.86) | 45(14.85) | 10.0553 | 0.0015 |
|  | High | 115(37.95) | 85(28.15) |  |  |
| TP | Normal | 188(62.05) | 217(71.85) | 6.5749 | 0.0103 |
|  | Low | 93(30.39) | 38(12.50) |  |  |
| GLB | Normal | 57(18.87) | 75(24.83) | 3.141 | 0.0763 |
|  | High | 246(86.62) | 221(79.50) |  |  |
| CREA | Normal | 38(13.38) | 57(20.50) | 5.0749 | 0.0243 |
|  | High | 227(75.42) | 193(63.70) |  |  |
| BUN | Normal | 74(24.58) | 110(36.30) | 9.7893 | 0.0018 |
|  | High | 3(0.98) | 2(0.67) |  |  |
| LDH | Normal | 302(99.02) | 295(99.33) | 0.1758 | 0.675 |
|  | High | 39(12.83) | 34(11.41) |  |  |
| CK | Normal | 265(87.17) | 264(88.59) | 0.2846 | 0.5937 |
|  | High | 46(17.83) | 34(14.11) |  |  |
| Hs_trop | Normal | 212(82.17) | 207(85.89) | 1.282 | 0.2575 |
|  | High | 181(74.79) | 161(75.23) |  |  |
| CK-MB | Normal | 61(25.21) | 53(24.77) | 0.0117 | 0.9137 |
|  | High | 1(0.50) | 1(0.56) |  |  |
| Ferrin | Normal | 198(99.50) | 177(99.44) | 0.0063 | 0.9369 |
|  | High | 139(45.87) | 179(60.47) |  |  |
| Na | Normal | 164(54.13) | 117(39.53) | 12.8126 | 0.0003 |
|  | Low | 173(57.28) | 184(61.33) |  |  |
| Ka | Normal | 129(42.72) | 116(38.67) | 1.0221 | 0.312 |
|  | Low | 73(24.17) | 80(27.12) |  |  |
| Ca | Normal | 229(75.83) | 215(72.88) | 0.6797 | 0.4097 |
|  | Low | 191(65.41) | 198(67.58) |  |  |
| Mg | Normal | 101(34.59) | 95(32.42) | 0.3079 | 0.579 |
|  | Low | 113(37.79) | 101(34.47) |  |  |
| phosphorus | Normal | 186(62.21) | 192(65.53) | 0.7074 | 0.4003 |
|  | Low | 124(58.22) | 164(79.23) | 21.5045 | <0.0001 |
| APTT | Normal | 55(18.15) | 88(29.53) |  |  |
|  | High | 248(81.85) | 210(70.47) | 10.7274 | 0.0011 |
| D_dimer | Normal | 5(1.75) | 5(1.84) |  |  |
|  | High | 281(98.25) | 267(98.16) | 0.0064 | 0.9362 |
| FIB | Normal | 268(88.74) | 233(78.19) |  |  |
|  | High | 34(11.26) | 65(21.81) | 12.1261 | 0.0005 |
| INR | Normal | 287(94.72) | 264(89.49) |  |  |
|  | High | 16(5.28) | 31(10.51) | 5.6413 | 0.0175 |
| PT | Normal | 264(87.13) | 253(85.76) |  |  |
|  | High | 39(12.87) | 42(14.24) | 0.2382 | 0.6255 |
| TT | Normal | 62(20.46) | 52(17.51) |  |  |
|  | High | 241(79.54) | 245(82.49) | 0.8502 | 0.3565 |
| Type | Mild | 36(11.76) | 25(8.22) |  |  |
|  | Moderate | 107(34.97) | 99(32.57) |  |  |
|  | Severe | 112(36.60) | 98(32.24) |  |  |
|  | Critical | 51(16.67) | 82(26.97) | 6.9862 | 0.0082 |

**Table S2**. Univeriate Cox regression before PSM

| Index | HR(95%CI) | P value |
| --- | --- | --- |
| Age | 5.472(2.822~10.609) | <0.0001 |
| CRP/PNI | 2.550(1.583~4.109) | 0.0001 |
| SFTSV RNA | 8.852(4.433~17.676) | <0.0001 |
| CK | 5.660(1.391~23.027) | 0.0150 |
| Hyper_trop | 7.274(1.786~29.631) | 0.0056 |
| CK-MB | 2.965(1.828~4.809) | <0.0001 |
| Mg | 1.718(1.094~2.698) | 0.0187 |
| CA | 2.703(1.349~5.414) | 0.0050 |
| PT | 2.995(1.856~4.832) | <0.0001 |
| TT | 2.993(1.303~6.877) | 0.0098 |

**Table S3**. Baseline features of SFTS patients in the PSM cohort

| **Index** | **Groups** | **Alive** | **Dead** | **X^2^ value** | **P value** |
| --- | --- | --- | --- | --- | --- |
| Sex | Female | 59(46.09) | 8(30.77) |  |  |
|  | Male | 69(53.91) | 18(69.23) | 2.0648 | 0.1507 |
| Age | ≤60 years | 62(48.44) | 3(11.54) |  |  |
|  | >60 years | 66(51.56) | 23(88.46) | 12.0623 | 0.0005 |
| smoking | No | 65(67.71) | 10(71.43) |  |  |
|  | Yes | 31(32.29) | 4(28.57) | 0.0779 | 0.7801 |
| alcohol | No | 70(77.78) | 10(71.43) |  |  |
|  | Yes | 20(22.22) | 4(28.57) | 0.2751 | 0.5999 |
| CRP/PNI | Low | 69(53.91) | 8(30.77) |  |  |
|  | High | 59(46.09) | 18(69.23) | 4.6274 | 0.0315 |
| FSTS V RNA | Low | 53(42.40) | 2(7.69) |  |  |
|  | High | 72(57.60) | 24(92.31) | 11.1964 | 0.0008 |
| CRP | Normal | 73(57.03) | 5(19.23) |  |  |
|  | High | 55(42.97) | 21(80.77) | 12.3535 | 0.0004 |
| PCT | Normal | 34(26.56) | 3(11.54) |  |  |
|  | High | 94(73.44) | 23(88.46) | 2.6723 | 0.1021 |
| WBC | Normal | 28(21.88) | 4(15.38) |  |  |
|  | Abnormal | 100(78.13) | 22(84.62) | 0.553 | 0.4571 |
| RBC | Normal | 111(86.72) | 23(88.46) |  |  |
|  | Low | 17(13.28) | 3(11.54) | 0.0581 | 0.8095 |
| HGB | Normal | 98(77.17) | 21(80.77) |  |  |
|  | Low | 29(22.83) | 5(19.23) | 0.1622 | 0.6872 |
| PLT | >50G/L | 26(20.31) | 7(26.92) |  |  |
|  | 30-50 | 46(35.94) | 10(38.46) |  |  |
|  | <30G/L | 56(43.75) | 9(34.62) | 0.8973 | 0.3435 |
| Lym | Normal | 12(9.38) | 1(3.85) |  |  |
|  | Abnormal | 116(90.63) | 25(96.15) | 0.8547 | 0.3552 |
| Mon | Normal | 69(53.91) | 10(38.46) |  |  |
|  | Abnormal | 59(46.09) | 16(61.54) | 2.0634 | 0.1509 |
| Neu | Normal | 40(31.25) | 10(38.46) |  |  |
|  | Abnormal | 88(68.75) | 16(61.54) | 0.5126 | 0.474 |
| ALT | Normal | 19(14.84) | 4(15.38) |  |  |
|  | High | 109(85.16) | 22(84.62) | 0.005 | 0.9438 |
| AST | Normal | 3(2.34) | 1(3.85) |  |  |
|  | High | 125(97.66) | 25(96.15) | 0.1928 | 0.6606 |
| GGT | Normal | 65(50.78) | 10(38.46) |  |  |
|  | High | 63(49.22) | 16(61.54) | 1.3129 | 0.2519 |
| ALP | Normal | 112(88.19) | 23(88.46) |  |  |
|  | High | 15(11.81) | 3(11.54) | 0.0015 | 0.9687 |
| TBIL | Normal | 116(90.63) | 22(84.62) |  |  |
|  | High | 12(9.38) | 4(15.38) | 0.8383 | 0.3599 |
| TP | Normal | 37(28.91) | 10(38.46) |  |  |
|  | Low | 91(71.09) | 16(61.54) | 0.9305 | 0.3347 |
| ALB | Normal | 25(19.53) | 8(30.77) |  |  |
|  | Low | 103(80.47) | 18(69.23) | 1.621 | 0.203 |
| GLB | Normal | 102(80.95) | 19(73.08) |  |  |
|  | High | 24(19.05) | 7(26.92) | 0.8234 | 0.3642 |
| CREA | Normal | 103(80.47) | 13(50.00) |  |  |
|  | High | 25(19.53) | 13(50.00) | 10.7937 | 0.001 |
| BUN | Normal | 87(67.97) | 21(80.77) |  |  |
|  | High | 41(32.03) | 5(19.23) | 1.6903 | 0.1936 |
| LDH | Normal | 2(1.56) | 0(0.00) |  |  |
|  | High | 126(98.44) | 26(100.00) | 0.4116 | 0.5212 |
| CK | Normal | 13(10.16) | 0(0.00) |  |  |
|  | High | 115(89.84) | 26(100.00) | 2.8841 | 0.0895 |
| Hs_trop | Normal | 16(15.24) | 1(4.35) |  |  |
|  | High | 89(84.76) | 22(95.65) | 1.9428 | 0.1634 |
| CK-MB | Normal | 73(78.49) | 12(66.67) |  |  |
|  | High | 20(21.51) | 6(33.33) | 1.1763 | 0.2781 |
| Ferrin | Normal | 2(2.41) | 0(0.00) |  |  |
|  | High | 81(97.59) | 14(100.00) | 0.3445 | 0.5573 |
| Na | Normal | 56(43.75) | 9(34.62) |  |  |
|  | Low | 72(56.25) | 17(65.38) | 0.7392 | 0.3899 |
| Ka | Normal | 76(59.84) | 14(53.85) |  |  |
|  | Low | 51(40.16) | 12(46.15) | 0.3204 | 0.5714 |
| Ca | Normal | 30(23.62) | 3(12.00) |  |  |
|  | Low | 97(76.38) | 22(88.00) | 1.6599 | 0.1976 |
| Mg | Normal | 84(66.67) | 11(44.00) |  |  |
|  | Low | 42(33.33) | 14(56.00) | 4.5936 | 0.0321 |
| phosphorus | Normal | 42(33.87) | 9(36.00) |  |  |
|  | Low | 82(66.13) | 16(64.00) | 0.0419 | 0.8378 |
| APTT | Normal | 31(24.22) | 1(3.85) |  |  |
|  | High | 97(75.78) | 25(96.15) | 5.4486 | 0.0196 |
| D_dimer | Normal | 3(2.46) | 0(0.00) |  |  |
|  | High | 119(97.54) | 24(100.00) | 0.6025 | 0.4376 |
| FIB | Normal | 109(85.16) | 19(73.08) |  |  |
|  | High | 19(14.84) | 7(26.92) | 2.247 | 0.1339 |
| INR | Normal | 122(95.31) | 20(76.92) |  |  |
|  | High | 6(4.69) | 6(23.08) | 10.1712 | 0.0014 |
| PT | Normal | 114(89.06) | 19(73.08) |  |  |
|  | High | 14(10.94) | 7(26.92) | 4.6891 | 0.0304 |
| TT | Normal | 20(15.63) | 3(11.54) |  |  |
|  | High | 108(84.38) | 23(88.46) | 0.2841 | 0.594 |
| Type | Mild | 12(9.38) | 0(0.00) |  |  |
|  | Moderate | 44(34.38) | 2(7.69) |  |  |
|  | Severe | 46(35.94) | 14(53.85) |  |  |
|  | Critical | 26(20.31) | 10(38.46) | 10.912 | 0.001 |

**Table S4**. Clinical correlation of CRP/PNI in individuals with SFTS after PSM

| **Index** | **Groups** | **Low CRP/PNI** | **High CRP/PNI** | **X^2^ value** | **P value** |
| --- | --- | --- | --- | --- | --- |
| Sex | Female | 33(42.86) | 34(44.16) |  |  |
|  | Male | 44(57.14) | 43(55.84) | 0.0264 | 0.8709 |
| Age | ≤60 years | 37(48.05) | 28(36.36) |  |  |
|  | >60 years | 40(51.95) | 49(63.64) | 2.1563 | 0.1420 |
| smoking | No | 39(68.42) | 36(67.92) |  |  |
|  | Yes | 18(31.58) | 17(32.08) | 0.0031 | 0.9554 |
| alcohol | No | 41(78.85) | 39(75.00) |  |  |
|  | Yes | 11(21.15) | 13(25.00) | 0.2167 | 0.6416 |
| SFTSV RNA | Low | 27(36.00) | 28(36.84) |  |  |
|  | High | 48(64.00) | 48(63.16) | 0.0116 | 0.9144 |
| PCT | Normal | 18(23.38) | 19(24.68) |  |  |
|  | High | 59(76.62) | 58(75.32) | 0.0356 | 0.8504 |
| WBC | Normal | 13(16.88) | 19(24.68) |  |  |
|  | Abnormal | 64(83.12) | 58(75.32) | 1.4201 | 0.2334 |
| RBC | Normal | 67(87.01) | 67(87.01) |  |  |
|  | Low | 10(12.99) | 10(12.99) | 0.0000 | 1.0000 |
| HGB | Normal | 59(76.62) | 60(78.95) |  |  |
|  | Low | 18(23.38) | 16(21.05) | 0.1195 | 0.7296 |
| PLT | >50G/L | 17(22.08) | 16(20.78) |  |  |
|  | 30-50 | 28(36.36) | 28(36.36) |  |  |
|  | <30G/L | 32(41.56) | 33(42.86) | 0.0435 | 0.8348 |
| Mon | Normal | 38(49.35) | 41(53.25) |  |  |
|  | Abnormal | 39(50.65) | 36(46.75) | 0.2339 | 0.6286 |
| Neu | Normal | 21(27.27) | 29(37.66) |  |  |
|  | Abnormal | 56(72.73) | 48(62.34) | 1.8954 | 0.1686 |
| ALT | Normal | 12(15.58) | 11(14.29) |  |  |
|  | High | 65(84.42) | 66(85.71) | 0.0511 | 0.8211 |
| AST | Normal | 3(3.90) | 1(1.30) |  |  |
|  | High | 74(96.10) | 76(98.70) | 1.0267 | 0.3109 |
| GGT | Normal | 37(48.05) | 38(49.35) |  |  |
|  | High | 40(51.95) | 39(50.65) | 0.026 | 0.8719 |
| ALP | Normal | 66(85.71) | 69(90.79) |  |  |
|  | High | 11(14.29) | 7(9.21) | 0.9491 | 0.3300 |
| TBIL | Normal | 70(90.91) | 68(88.31) |  |  |
|  | High | 7(9.09) | 9(11.69) | 0.2790 | 0.5974 |
| TP | Normal | 25(32.47) | 22(28.57) |  |  |
|  | Low | 52(67.53) | 55(71.43) | 0.2756 | 0.5996 |
| GLB | Normal | 62(82.67) | 59(76.62) |  |  |
|  | High | 13(17.33) | 18(23.38) | 0.8547 | 0.3552 |
| CREA | Normal | 59(76.62) | 57(74.03) |  |  |
|  | High | 18(23.38) | 20(25.97) | 0.1397 | 0.7085 |
| BUN | Normal | 57(74.03) | 51(66.23) |  |  |
|  | High | 20(25.97) | 26(33.77) | 1.1159 | 0.2908 |
| LDH | Normal | 1(1.30) | 1(1.30) |  |  |
|  | High | 76(98.70) | 76(98.70) | 0.0000 | 1.0000 |
| CK | Normal | 9(11.69) | 4(5.19) |  |  |
|  | High | 68(88.31) | 73(94.81) | 2.1004 | 0.1473 |
| Hs_trop | Normal | 8(12.12) | 9(14.52) |  |  |
|  | High | 58(87.88) | 53(85.48) | 0.1592 | 0.6899 |
| CK-MB | Normal | 45(76.27) | 40(76.92) |  |  |
|  | High | 14(23.73) | 12(23.08) | 0.0065 | 0.9355 |
| Ferrin | Normal | 1(2.17) | 1(1.96) |  |  |
|  | High | 45(97.83) | 50(98.04) | 0.0054 | 0.9412 |
| Na | Normal | 31(40.26) | 34(44.16) |  |  |
|  | Low | 46(59.74) | 43(55.84) | 0.2396 | 0.6245 |
| Ka | Normal | 46(60.53) | 44(57.14) |  |  |
|  | Low | 30(39.47) | 33(42.86) | 0.1808 | 0.6707 |
| Ca | Normal | 16(20.78) | 17(22.67) |  |  |
|  | Low | 61(79.22) | 58(77.33) | 0.0796 | 0.7778 |
| Mg | Normal | 50(65.79) | 45(60.00) |  |  |
|  | Low | 26(34.21) | 30(40.00) | 0.5423 | 0.4615 |
| phosphorus | Normal | 29(38.67) | 22(29.73) |  |  |
|  | Low | 46(61.33) | 52(70.27) | 1.3215 | 0.2503 |
| APTT | Normal | 14(18.18) | 18(23.38) |  |  |
|  | High | 63(81.82) | 59(76.62) | 0.6311 | 0.4269 |
| D_dimer | Normal | 2(2.70) | 1(1.39) |  |  |
|  | High | 72(97.30) | 71(98.61) | 0.313 | 0.5759 |
| FIB | Normal | 64(83.12) | 64(83.12) |  |  |
|  | High | 13(16.88) | 13(16.88) | 0.0000 | 1.0000 |
| INR | Normal | 71(92.21) | 71(92.21) |  |  |
|  | High | 6(7.79) | 6(7.79) | 0.0000 | 1.0000 |
| PT | Normal | 67(87.01) | 66(85.71) |  |  |
|  | High | 10(12.99) | 11(14.29) | 0.0551 | 0.8144 |
| TT | Normal | 10(12.99) | 13(16.88) |  |  |
|  | High | 67(87.01) | 64(83.12) | 0.4600 | 0.4976 |
| Type | Mild | 9(11.69) | 3(3.90) |  |  |
|  | Moderate | 19(24.68) | 27(35.06) |  |  |
|  | Severe | 31(40.26) | 29(37.66) |  |  |
|  | Critical | 18(23.38) | 18(23.38) | 0.1298 | 0.7187 |

**Table S5**. Univeriate Cox regression after PSM

| Index | HR(95%CI) | P value |
| --- | --- | --- |
| Age | 6.247(1.875~20.814) | 0.0028 |
| CRP/PNI | 2.437(1.059~5.606) | 0.0362 |
| RNA | 7.241(1.711~30.640) | 0.0072 |
| Mg | 2.283(1.036~5.030) | 0.0406 |
| PT | 2.549(1.071~6.064) | 0.0344 |
